# Monitoring Canada’s Health Care Polycrisis Through News Headline Analysis: Infodemiology Study

**DOI:** 10.2196/99655

**Published:** 2026-10-29

**Authors:** John Weirstrass Muteba Mwamba, Vijay Mago

**Affiliations:** 1 School of Health Policy and Management Faculty of Health York University Toronto, ON Canada

**Keywords:** infoveillance, Granger causality, health care policy, natural language processing, polycrisis, sentiment analysis

## Abstract

**Background:**

The health care polycrisis in Canada comprises several concurrent crises, namely workforce shortages; food insecurity; housing stress; and health inequities affecting Indigenous peoples, low-income families, and racialized communities. A scalable infoveillance system is needed to monitor these related but distinct crises concurrently using natural language processing (NLP) and statistical forecasting.

**Objective:**

This study applied a multimethod NLP pipeline to characterize discourse dynamics, causal interdependencies among crisis themes, and the responsiveness of policy-making to those dynamics within Canada’s health care system from 2020 to 2025.

**Methods:**

We used an NLP pipeline to examine the language and sentiment of 6757 online news headlines regarding Canada’s health care system over approximately 5 years (2020-2025), using 2 methods for sentiment analysis: a fine-tuned BERT (Bidirectional Encoder Representations from Transformers) model (test accuracy of 0.97) and VADER (Valence Aware Dictionary and Sentiment Reasoner) for valence and subjectivity. We used latent Dirichlet allocation to identify topics within the headlines, then applied time series analysis, using both ARIMA (autoregressive integrated moving average) and ETS (error, trend, and seasonality) methods, to predict trends in each topic. Finally, we used Granger causality testing to determine whether past values of one theme could predict future values of another.

**Results:**

Seven major discourse topics emerged, of which 5 were retained for the surveillance analysis. The health care system crisis theme was the most prevalent (1200/6757, 17.8% of all headlines), followed by economic determinants of health (1017/6757, 15.1%), housing as a health determinant (958/6757, 14.2%), food insecurity health impact (929/6757, 13.7%), and social inequality health effects (699/6757, 10.3%). Strong statistical evidence showed that the volume of discourse around food insecurity health impact was temporally predictive of subsequent discourse volume around housing as a health determinant (*F*_1,54_=17.361; false discovery rate–adjusted *P*=.002). Sentiment analysis showed no statistically significant change after any policy intervention (Cohen *d* <0.2), and the 12-month forecasts were flat or slightly lower for 4 of the 5 retained themes and rose only for health care system crisis (all 95% CIs included zero).

**Conclusions:**

These findings illustrate the complexity of issues currently facing Canada and suggest that policymakers should consider implementing NLP-based infoveillance systems to monitor and analyze complex relationships between these crises. Further, our findings suggest a persistent policy perception gap with significant implications for designing health informatics surveillance systems.

## Introduction

Global health care systems face an unprecedented set of interrelated, simultaneous, and interactive crises. These crises include severe workforce shortages; economic instability; persistent and worsening social and economic disparities; and demographic pressures. These health care crises can no longer be viewed as discrete epidemiological events developing in isolation [1,2]. Rather, they represent part of a larger polycrisis: a type of event where multiple, systemic crises interact with one another and produce a total impact much greater in magnitude than if the individual crises were analyzed independently in isolation [3,4].

An important characteristic of a polycrisis is the development of upward-downward cascade failures, that is, when a major crisis develops in a key area (eg, housing or food insecurity), the downward trajectory in the population’s health status and the burden placed upon the health care delivery system will accelerate dramatically [5].

Canada’s health care system is currently experiencing a polycrisis. Canada faces 3 main, concurrent crises simultaneously: unprecedented levels of housing stress; record levels of food insecurity; and severe and growing workforce shortages within the health care system [6-8]. Each of these crises also results in disproportionate negative effects on Indigenous populations, low-income families, and racialized communities—contributing to historical health inequities [9]. Moreover, despite the obvious linkages between these crises, the majority of responses to date have consisted of independent policy actions across separate crisis themes without consideration for the causal relationships between the socioeconomic determinants of the population’s health and the health care services provided.

To manage a health care polycrisis effectively, we need to go beyond traditional epidemiologic surveillance methods used by virtually all nations. Traditional surveillance approaches are generally disease-specific and rely on historical data.

The unstructured text generated from online digital discourse, including that derived from news media, contains a vast amount of information that reflects the interaction of the various crisis themes in real time [10]. While natural language processing (NLP) techniques, specifically topic modeling and sentiment analysis using transformer models (such as BERT [Bidirectional Encoder Representations from Transformers]), have demonstrated success in identifying public health indicators [11,12], there is a substantial lack of evidence demonstrating their application to monitor the health care polycrisis in Canada. Virtually all previous infoveillance (in this study, we use the term infoveillance to refer specifically to the use of publicly available digital data, including online news and search behavior [10], to monitor population-level health trends, and we use this term consistently rather than the related but distinct terms digital surveillance and infosurveillance) studies in Canada have focused almost exclusively on single events (eg, the COVID-19 pandemic) [13] and not on the comprehensive network of food insecurity, housing, financial difficulties, and systemic inadequacies in the delivery of health care simultaneously.

Therefore, a sizeable gap in the literature exists: a robust, multimethod infoveillance tool capable of monitoring the entire interconnected health system crises in Canada is nonexistent. In particular, the causal structure linking public discourse associated with these different crisis themes has not been quantitatively modeled, and neither has the public sentiment toward health care been statistically correlated to the timing of policy actions taken to address the health care polycrisis.

This study seeks to fill this void by creating a scalable multimethod NLP pipeline to identify the causal relationship between public discourse and the various components of the health care polycrisis in Canada between 2020 and 2025. We propose to achieve this objective through the application of a variety of complementary analytical tools, including transformer-based sentiment analysis; latent Dirichlet allocation (LDA)–based topic modeling; Granger causality-based econometric testing for causality; and ARIMA (autoregressive integrated moving average)/ETS (error, trend, and seasonality)–based statistical forecasting. Through our proposed pipeline, we plan to model the public discourse in Canadian online news headlines. Ultimately, our objective is to predict the future trends of the critical crisis themes and evaluate the responsiveness of the policy-making process to the evolving online public discourse surrounding the delivery of health care.

## Methods

### Overview

The proposed health care infoveillance tool comprised 4 sequential analytical phases shown in Figure 1.

**Figure 1:**
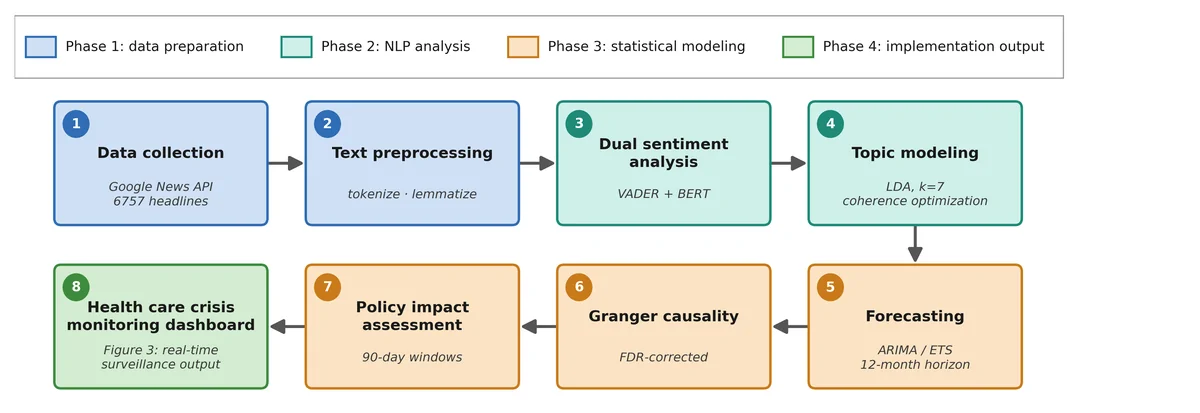
The multimethod NLP digital infoveillance pipeline, with each phase of analysis represented by a different color. Blue represents the data preparation phase; teal represents both sentiment and topic modeling phases within the NLP analysis phase; orange represents forecasting, causal network analysis, and policy impact assessment within the statistical modeling phase; and green represents implementation outputs. Numbered boxes indicate the processing order. The ultimate goal of this study is to develop an analytical health care crisis monitoring prototype (see Figure 3). ARIMA: autoregressive integrated moving average; BERT: bidirectional encoder representations from transformers; ETS: error, trend, and seasonality; FDR: false discovery rate; LDA: latent Dirichlet allocation; NLP: natural language processing; VADER: Valence Aware Dictionary and Sentiment Reasoner.

### Data Collection and Preprocessing

We created a digital infoveillance tool for health care through the use of publicly available online news headlines. Eysenbach [10] demonstrated that the number of news headlines is large in scale and can be used as an indicator of the most prominent issues or concerns of a population. For our analysis, we accessed online news headlines using the Google News API (GoogleNews Python library), with 12 different search terms over 5 different crisis themes: housing as a determinant of health, health care system collapse, food insecurity, financial hardship, and social inequality. The time frame for which data was collected began on October 17, 2020, and ended on September 28, 2025. In total, there were 6757 unique English-language headlines posted online by Canadian news media outlets. Each of the records included information regarding the headline itself, the date it was published online, the name of the publishing outlet, and the terms that generated the headline.

The 12 search terms that produced the corpus were the following: “Canada polycrisis,” “Canada’s economic crisis,” “Canada’s affordability crisis,” “Canada’s healthcare crisis,” “Collapse of the Canadian Healthcare System,” “Crisis in Ontario Healthcare,” “Privatizing Healthcare in Canada,” “Income Inequality in Canada,” “Food Insecurity in Canada,” “Racialized Communities’ Access to Healthcare in Canada,” “Affordability Crisis in Housing in Canada,” and “Homeless Crisis in Canada,” all limited to English-language articles and to the Canada region. The data were sourced from 259 unique news outlets; although there was an evident concentration of reporting, no one outlet represented more than half of all media content. Headlines from CBC (Canadian Broadcasting Corporation) made up 13.3% (897/6757) of the total headlines, while headlines from Global News made up 4.7% (320/6757), those from the Fraser Institute made up 3.7% (248/6757), those from the Financial Post made up 2.6% (174/6757), and those from The Conversation made up 2.5% (170/6757). Together, these top 5 outlets accounted for approximately 26.8% (1809/6757) of all headline content, while the top 10 outlets collectively accounted for 37.8% (2554/6757) of headline content. Although the retrieval of headlines via Google’s News aggregation and ranking algorithms may be less prone to sampling error than using either a truly random or a comprehensive set of Canadian news stories, this approach nonetheless introduces its own potential sources of sampling bias [14]. These sources include the composition of outlets included in the corpus and the coverage of the search terms used in the retrieval process.

A typical NLP pipeline was applied for preprocessing. In addition to converting all characters in each string of text to lowercase, all HTML tag elements were eliminated, as well as URLs and special characters. Each string of text was then broken down into individual words through the use of Natural Language Toolkit (NLTK). Using WordNet, the root form (or base) of each word (ie, “running” becomes “run”) was identified. As such, common English words (stopwords) are typically eliminated in an effort to remove noise from data. However, in this case, it is possible some of those English stopwords would be important to include because they were related to themes such as health or health care, Canada or Canadian, systems, and crises. All strings with fewer than 3 characters were removed from the dataset.

This study used only publicly available news headline titles collected from the Google News API. There were no humans involved in this study, nor were there biological samples or personally identifiable information. Therefore, Ethics Board Review was not needed at York University.

### Sentiment Surveillance

VADER (Valence Aware Dictionary and Sentiment Reasoner) [15] was run on all 6757 raw online news headline data points to create compound scores ranging from –1 to +1. The headlines were then categorized based on the VADER scores. If the VADER score was greater than or equal to 0.05, it was considered a positive headline. If the VADER score was less than or equal to –0.05, it was considered a negative headline. All other headlines were categorized as neutral. The monthly aggregation of these scores formed the VADER surveillance series.

The BERT-base-uncased [16] was fine-tuned for 3-class sentiment classification. To enhance computational efficiency, a stratified random sample of 3000 headlines was sampled from the total 6757-headline dataset. The stratified random sample was then divided into an 80% (2400/3000) holdout training set and a 20% (600/3000) held-out testing set. Stratification by class label was carried out at both partitions to preserve the original distribution of class labels. The original class labels for each headline were assigned using the same VADER classification models that were originally trained. Following fine-tuning, the model was run again but in inference mode over all 6757 headlines in the entire dataset. This run through the entire dataset generated the BERT sentiment scores shown in Figure 2 and the monitoring dashboard (Figure 3), and the evaluated model agreement with VADER. The hyperparameters chosen included 2 epochs, a batch size of 16, a learning rate of 2×10^-5^, the AdamW optimizer, and a max sequence length of 128. These hyperparameters are within acceptable ranges recommended for the BERT-base-uncased model on downstream classification tasks [16]. It is worth noting that a formal hyperparameter search was not conducted, and this is noted as a limitation. A continuous sentiment score was calculated as the difference in positive and negative sentiment probabilities from the softmax output.

**Figure 2:**
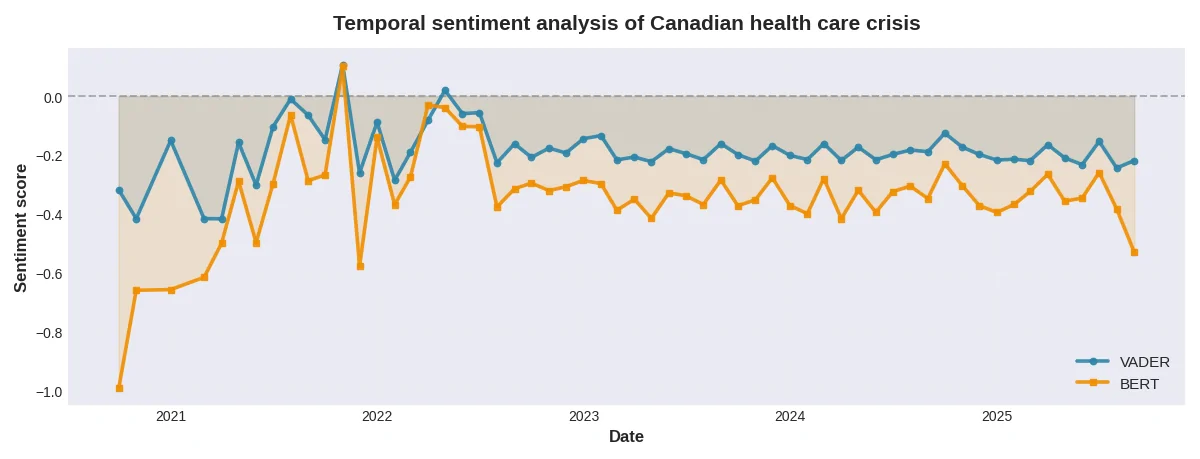
VADER and BERT monthly sentiment scores for Canadian health care polycrisis (October 2020 to September 2025). Both time series display consistent negative orientations and periodic peaks in negative intensity. Shaded areas extend from each series to zero. BERT: bidirectional encoder representations from transformers; VADER: Valence Aware Dictionary and Sentiment Reasoner.

**Figure 3:**
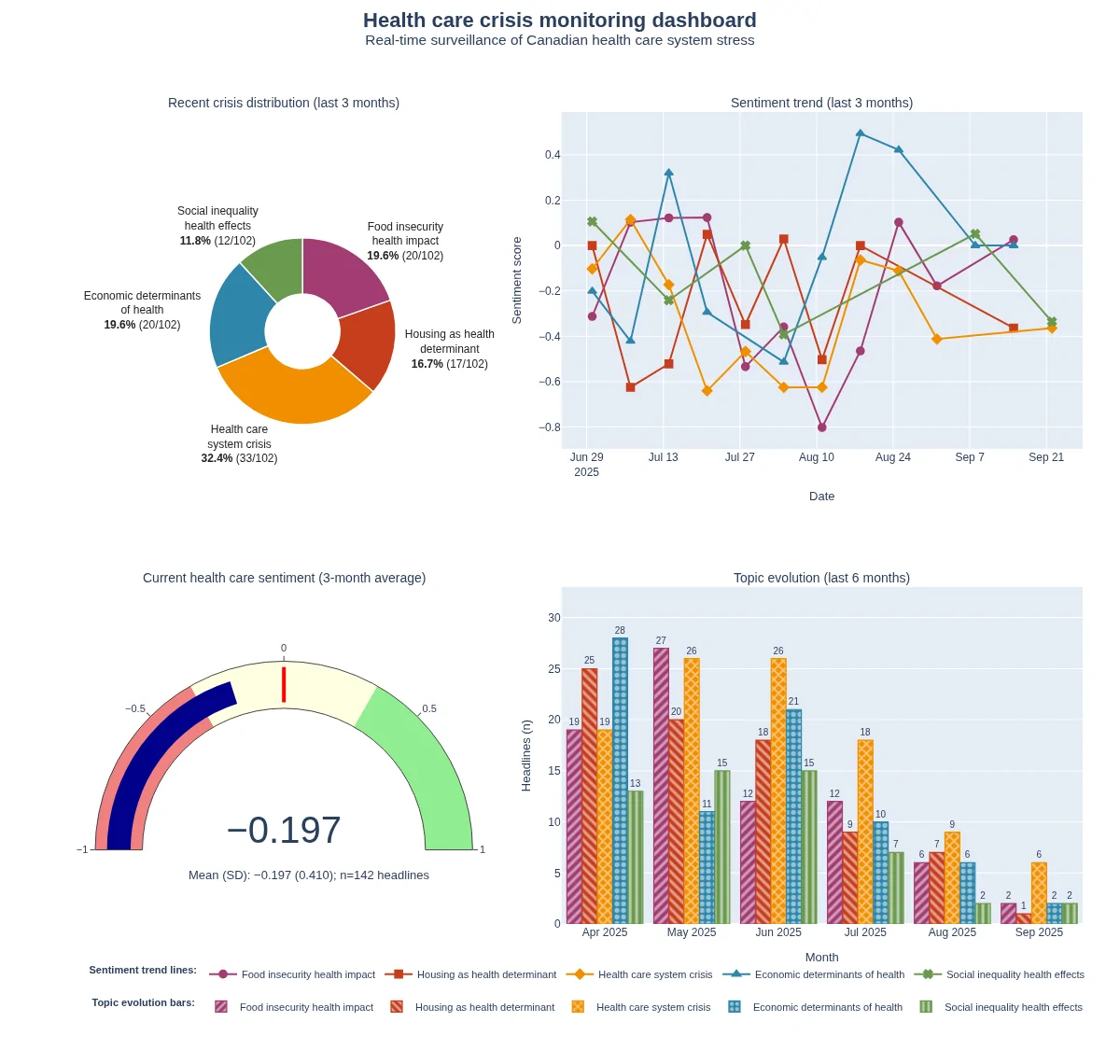
The prototype health care crisis monitoring dashboard that displays all monitoring results. At the top left, there is a donut chart illustrating the proportion of each crisis theme in the latest 3 months. At the top right, there are line charts of sentiment trends for each theme in the latest 3 months, with consistent colors used in the donut chart. The gauge indicator at the bottom left shows the rolling sentiment score averaged over the last 3 months (–0.197, SD 0.410; n=142 headlines), which is firmly negative. The bar chart at the bottom right shows how frequently each topic has been discussed each month over the last 6 months, with the aim of identifying topics with increasing discussion. This dashboard demonstrates that our pipeline can be used as a near-real-time health informatics surveillance tool.

### Topic Modeling

LDA [17] was applied to the preprocessed corpus. The vocabulary was filtered so that only those terms that appeared in more than 5 and fewer than half of the documents were included. A range of topic numbers (k=3, ..., 7) was tested to determine which number of topics provided the highest value of c_v_ coherence. The model was run with a total of 20 iterations, with the hyperparameters set to automatically estimate the Dirichlet parameters, and the random seed was set to 42. For each headline, the most likely topic was identified and assigned to it. The monthly topic frequencies were created [12].

First, we quantitatively evaluated the topic coherence using the c_v_ coherence measure [18]; the common values for coherence evaluation that have been reported in the literature when evaluating short informal texts (such as headlines, X [X Corp] posts, or forum postings) typically fall into the 0.4 to 0.6 range. The reason behind these lower ranges can be attributed to the fact that shorter texts provide fewer word co-occurrences relative to longer texts (for example, full articles or abstracts), which generally produce higher c_v_ measures. The c_v_ measure calculated for this dataset (c_v_=0.453) fits within the established acceptable range. In addition to representing some degree of true thematic overlap (as indicated by the presence of “care” among the keyword lists for more than one topic), this relatively low c_v_ score likely represents a portion of theme overlap.

The LDA was chosen over embedding-based alternatives such as BERTopic [19] and Top2Vec [20] due to its ability to generate fully interpretable word-topic and document-topic probability distributions, as well as its deterministic nature provided that a fixed random seed is used. This combination of features is important in the context of a surveillance system that needs to be auditable by health policy stakeholders.

### Time Series Forecasting

Each of the topic frequency time series was tested for stationarity using the augmented Dickey-Fuller test. Time series that failed the augmented Dickey-Fuller test were first-differenced to make them stationary. For each of the 5 primary topics, two models were compared: an ARIMA(1,1,1) and an ETS (additive trend and seasonal) model with period 12. The performance of each of these models was evaluated for the final 6 months of data available using both mean absolute error and root-mean-square error. The 12-month forecasts with 95% CIs reported in Figure 4 were generated with the ARIMA(1,1,1) model for all 5 topics; the ETS model was used only for the accuracy comparison.

**Figure 4:**
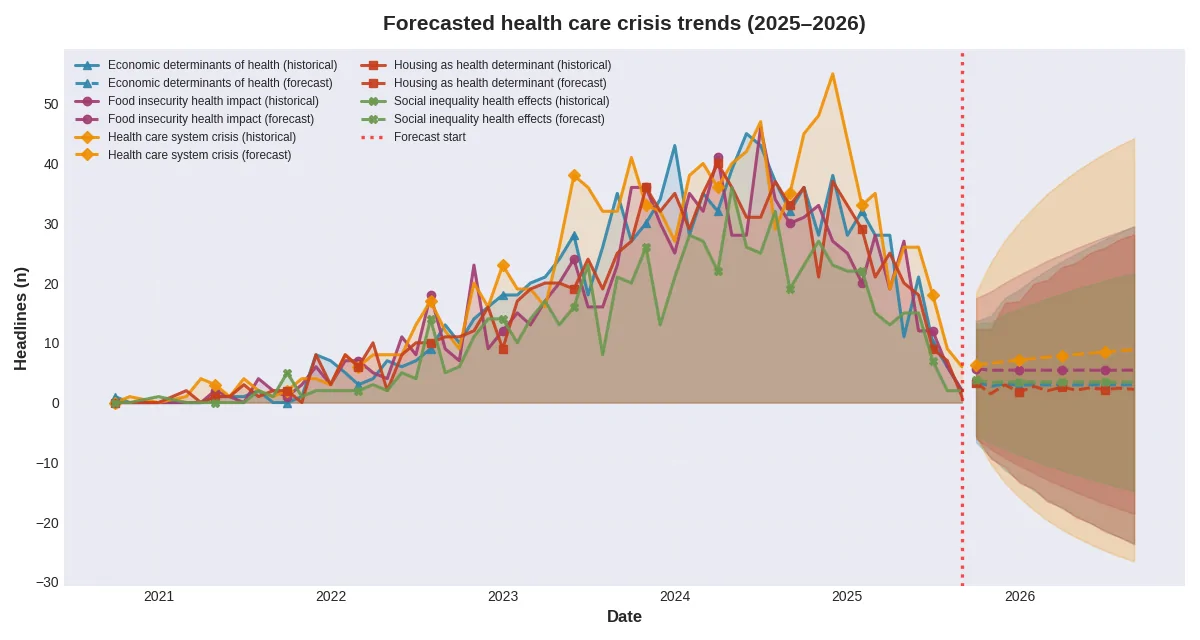
Historical monthly frequency of discourse and 12-month forecasts (ARIMA(1,1,1) model) with 95% CIs. Forecasts are flat or slightly lower for 4 topics and rise for health care system crisis; all 95% CIs include zero. ARIMA: autoregressive integrated moving average.

### Granger Causality Analysis

For each of the 20 pairs of primary topics, pairwise Granger causality tests [21,22] were carried out on the topic frequency time series from lag 1 through lag 6. Since our null hypothesis is that the topic frequency time series do not provide predictive information to each other, we are dealing with a multiple testing problem. Therefore, we used the corrected *P* values to account for this issue using Benjamini-Hochberg false discovery rate (FDR) at α=.05 [23]. In addition, all of our Granger causality results were operationalized to indicate whether or not prior discussion in 1 area provided statistically significant predictive information relative to another area (beyond what has already been observed within the prior discussion of the other area).

### Policy Intervention Analysis

To evaluate the impact of 8 policy interventions on crisis themes such as economic support, food security, health care privatization, workforce expansion, and housing-health integration, we examined the topic frequency time series for each intervention. We did this by calculating the symmetric difference of the topic frequency time series before and after each policy intervention for each type of intervention. To evaluate whether the changes in the topic frequency time series were due to chance, 2-tailed independent-samples *t* tests, Cohen d effect sizes, and 1000 resample bootstrap 95% CIs were calculated. To assess how long a policy intervention could sustain a positive sentiment, we defined sentiment sustainability as the amount of time that a topic frequency time series could stay above 0.05 units of the baseline (max 180 days). As with the Granger causality analysis, we also had multiple hypotheses to test (8 policy interventions) and therefore applied FDR correction to each of the tests. A full description of all 8 interventions, including type, date, rationale, and implementation score, is provided in Multimedia Appendix 1.

### Predictive Feature Analysis

To predict negative sentiment based on the features calendar month, year, and headline text length, a random forest classifier (100 trees, max depth 10, random seed 42) was trained. The importance of each feature was measured as the average reduction in Gini impurity when splitting the training data based on that feature. All analyses were carried out in Python (Python Software Foundation) using *pandas*, *NumPy*, *NLTK*, *Gensim*, *statsmodels*, *scikit-learn*, *PyTorch*, and *Hugging Face Transformers*. The random seed was applied worldwide, including to the stratified train or validation splits that were performed before BERT fine-tuning, and to both stages of LDA topic modeling. The ARIMA and ETS models, the augmented Dickey-Fuller test, the Granger causality tests, and the 2-tailed independent samples *t* test all use deterministic methods and therefore have no randomness or stochastic components; they required no seed. Additionally, we ran a robustness check on our Random Forest feature importance scores using Shapley additive explanations (SHAP) values [24] via a TreeExplainer since it has been shown that Gini Impurity is biased toward continuous and high cardinality features [25]. These results are presented in the Predictive Feature Analysis subsection of the Results section.

### Ethical Considerations

Our study consists solely of publicly available news headline titles. As such, we neither included nor referenced human participants, biological samples, nor personal identifying information. Consequently, ethics committee approval and/or participant consent were not necessary.

## Results

### Sentiment Surveillance

The fine-tuned BERT classifier performed with an accuracy of 0.97 (weighted *F*_1_-score=0.97) on the held-out test set (n=600) drawn from the 3000-headline stratified subsample described in the Methods section. On this test set, for the negative class, precision, recall, and *F*_1_-scores were all equal to 0.99; for the neutral class, precision was 1.00, recall was 0.93, and *F*_1_-score was 0.96; for the positive class, precision was 0.93, recall was 0.98, and *F*_1_-score was 0.95. Both VADER and BERT models indicate a generally negative monthly sentiment trajectory from 2020 to 2025, with dips in sentiment occurring during periods of crisis and a similar month-to-month pattern in the 2 models (r=0.81 across the 58 monthly means), although BERT scores were lower than VADER scores in 57 of the 58 months (mean difference –0.15), as shown in Figure 2.

### Topic Modeling: Thematic Structure of the Polycrisis

Using LDA, 7 topic clusters were chosen based upon a model fit (c_v_=0.453). Of these 7 topics, 5 could be substantively interpreted to form the core themes used for surveillance purposes; however, an additional two—affordable housing policy deficit (996/6757, 14.7%) and Canadian housing cost burden (958/6757, 14.2%)—represented overlapping themes associated with Canada’s housing crisis. These 2 topics have been removed from the causal analysis.

Table 1 reports that health care system crisis was the most common topic at 17.8% (1200/6757) of all headlines, followed by economic determinants of health at 15.1% (1017/6757); housing as a health determinant at 14.2% (958/6757), food insecurity and health impact at 13.7% (929/6757), and social inequality and health effects at 10.3% (699/6757). Health care system crisis had been covered more than any other single theme in the sample corpus we collected; yet, when we look at the 4 upstream social-determinant themes (economic determinants of health, housing as health determinant, food insecurity health impact, and social inequality health effects) together they account for the majority of all headlines (3603/6757, 53.3%), indicating that while news outlets in Canada are covering some of the immediate manifestations of this crisis in their coverage of health care system crisis; they are also framing much of the coverage through the lens of its drivers, which are identified by the Granger causality analysis reported in the Granger Causality Network subsection of this section.

**Table 1:** Emergent themes from health care polycrisis discourse, identified through LDA^a^ (c_v_=0.453, k=7). This depicts all 7 topics. The first 5 rows represent the main surveillance topics, with the last 2 rows reflecting the intersecting threads of Canada’s housing crisis, which we omitted from causal analyses.

Topic theme	Representative keywords	Values, n (%)
Food insecurity health impact	food, insecurity, care, economy, recession	929 (13.7)
Housing as health determinant	care, Ontario, recession, Ford, public	958 (14.2)
Health care system crisis	Ontario, care, hospital, privatization, doctor	1200 (17.8)
Economic determinants of health	care, housing, income, inequality, could	1017 (15.1)
Social inequality health effects	polycrisis, housing, global, say, care	699 (10.3)
Affordable housing policy deficit	housing, care, new, government, affordability	996 (14.7)
Canadian housing cost burden	housing, economic, care, say, Canadian	958 (14.2)
Total (all topics)	N/A^b^	6757 (100.0)

^a^LDA: latent Dirichlet allocation.

^b^N/A: not applicable.

### Time Series Forecasting

The results of the augmented Dickey-Fuller tests indicate that all 5 crisis themes we retained were found to be nonstationary (the values of the p-statistic, which ranged from 0.055 to 0.541). However, upon taking the first difference of each nonstationary time series, the resulting series were transformed into stationary time series. All time series received the same uniform treatment based on their p-statistics’ proximity to the 0.05 threshold value.

Therefore, it is assumed that this rule-based methodology reduced the potential for subjective judgments regarding stationarity based on individual cases. Regarding performance metrics for model fit, as shown in Table 2, the ETS model outperformed the ARIMA model for 3 crises: economic determinants of health, housing as a health determinant, and social inequality health effects. Conversely, the ARIMA model performed better than the ETS model for 2 crises: health care system crisis and food insecurity health impact. Figure 4 exhibits the historical monthly frequency of each theme and the 12-month forecasts with 95% CIs.

**Table 2:** Time series model performance by discourse theme (MAE^a^ and RMSE^b^ on the final 6 months of observed data).

Theme and model			MAE		RMSE
Economic determinants of health					
	ARIMA^c^	16.53		18.72	
	ETS^d,e^	11.77		13.14	
Health care system crisis					
	ARIMA^e^	20.26		21.71	
	ETS	27.21		28.34	
Food insecurity health impact					
	ARIMA^e^	13.32		15.35	
	ETS	16.79		19.20	
Housing as a health determinant					
	ARIMA	12.17		14.59	
	ETS^e^	6.83		8.51	
Social inequality health effects					
	ARIMA	10.64		12.00	
	ETS^e^	6.66		7.62	

^a^MAE: mean absolute error.

^b^RMSE: root-mean-square error.

^c^ARIMA: autoregressive integrated moving average.

^d^ETS: error, trend, and seasonality.

^e^Best-performing model for that theme.

### Granger Causality Network

The FDR-corrected Granger causality results reported in Table 3 below show that food insecurity and its health effects discourse were Granger-caused by the economic determinant of health discourse (*F*_1,54_=7.60, FDR *P* value=.024).

This relationship supports an upstream ordering where coverage of economic hardship is first followed by coverage of food insecurity and then followed by coverage of housing as a health determinant and social inequality health effects. It can therefore be interpreted as an association in discourse over time. However, it would be incorrect to infer that economic hardship causes food insecurity and/or housing stress in the population. The results also show that health care system crisis discourse Granger-caused both the housing as health determinant discourse (*F*_1,54_=5.95, FDR *P* value=.047) and the social inequality health effects discourse (*F*_1,54_=6.16, FDR *P* value=.043). Of these 5 relationships, 2 (food insecurity health impact to housing as health determinant; economic determinants of health to food insecurity health impact) retained their FDR-significance across all 6 lags, while the other 3 either diminished or became insignificant at some of the later lags, indicating that the strength of the causal signal for each of those relationships is greatest in the immediate period. Figure 5 shows the complete network of significant relationships across lags 1 to 6.

**Table 3:** FDR^a^-corrected Granger causality results (lag 1). All listed relationships are significant at α_FDR_=.05.

Cause theme	Effect theme	*F* test (*df*)	FDR *P* value
Food insecurity health impact	Housing as health determinant	17.361 (1, 54)	.002
Food insecurity health impact	Social inequality health effects	9.380 (1, 54)	.015
Economic determinants of health	Food insecurity health impact	7.601 (1, 54)	.024
Health care system crisis	Social inequality health effects	6.164 (1, 54)	.043
Health care system crisis	Housing as health determinant	5.954 (1, 54)	.047

^a^FDR: false discovery rate.

**Figure 5:**
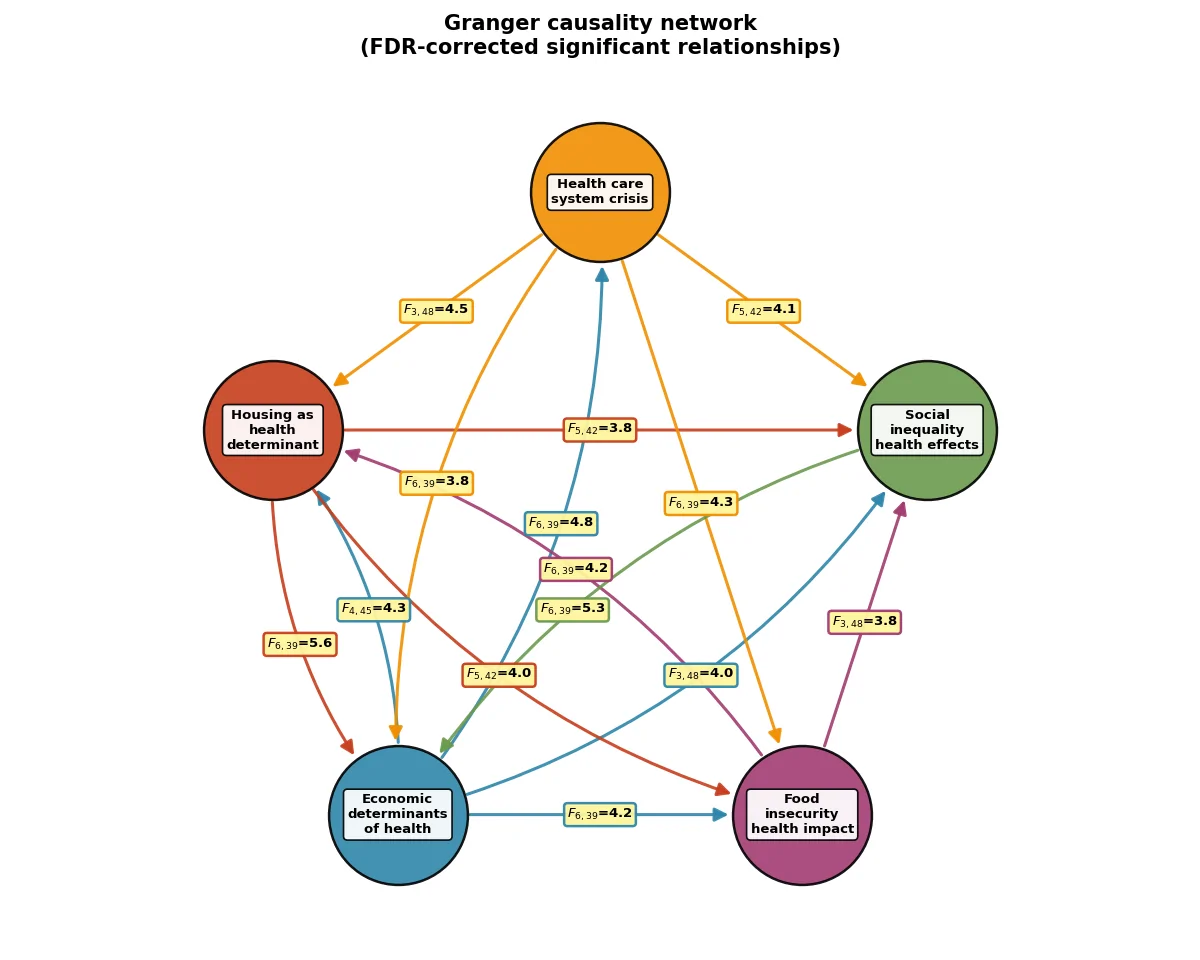
The Granger causality network represents all of the relationships between each of the themes identified within the Canadian health care polycrisis. Arrows point from the Granger-causing theme to the predicted theme; each label gives the F statistic with its degrees of freedom at the highest significant lag. In contrast to the results reported in Table 3 (which only included results for lag=1), Figure 5 includes every possible relationship between 2 themes that were statistically significant using an FDR-corrected F-test at some level of lag from 1 to 6. Therefore, while Figure 5 shows the directional relationships at specific levels of lag, it does represent a summary of the entire network over time rather than being limited to just one single level of lag. The node with the most edges connecting other nodes in Figure 5 is economic determinants of health (7), followed by housing as a health determinant (6); the other 3 themes have 5 each. FDR: false discovery rate.

### Policy Intervention Analysis

No intervention achieved statistical significance after applying FDR adjustment, with all interventions resulting in very small effect sizes (Cohen *d* <0.2). Economic support interventions resulted in the greatest absolute change (–0.056), with a small effect size (–0.128), and the most long-lasting impact (28 days). The only intervention type to cause an increase in positive sentiment was health care privatization. It produced a small absolute change (0.028) and a small effect size (0.064). It lasted only 1 day before returning to the baseline level. Workforce expansion produced almost no change (–0.004), with a negligible effect size (–0.010), despite having a higher implementation score (0.60) than Economic Support Interventions (0.20). The results are summarized in Table 4.

This can also be observed in Figure 6, where each colored vertical line represents a given policy intervention date overlaid on the continuous VADER and BERT sentiment scores. There is no observable change in trajectory in either sentiment score at any of these points. The baseline sentiment was continuously negative (mean VADER compound score –0.19, SD 0.43, across all 6757 headlines in the full sample period).

Both VADER and BERT scores remained negative in almost all months and fluctuated erratically, with no sustained trend, regardless of any intervention events. The BERT scores were lower than the VADER scores (by 0.15 on average), reflecting the negative nuances captured by the transformer model in the headlines. This further confirmed that the dates of policy interventions, as implemented and communicated to the public, do not appear to have made any significant impact on the dominant crisis narrative reflected in Canadian health news headlines.

**Table 4:** The impact of each type of policy intervention.

Policy type	Delta sentiment	Cohen *d*	Sustainability (days)	Implementation score
Economic support	–0.056	–0.128	28	0.20
Food security	–0.056	–0.122	10	0.40
Privatization	+0.028	+0.064	1	0.80
Workforce expansion	–0.004	–0.010	3	0.60
Housing integration	–0.017	–0.039	5	0.55

**Figure 6:**
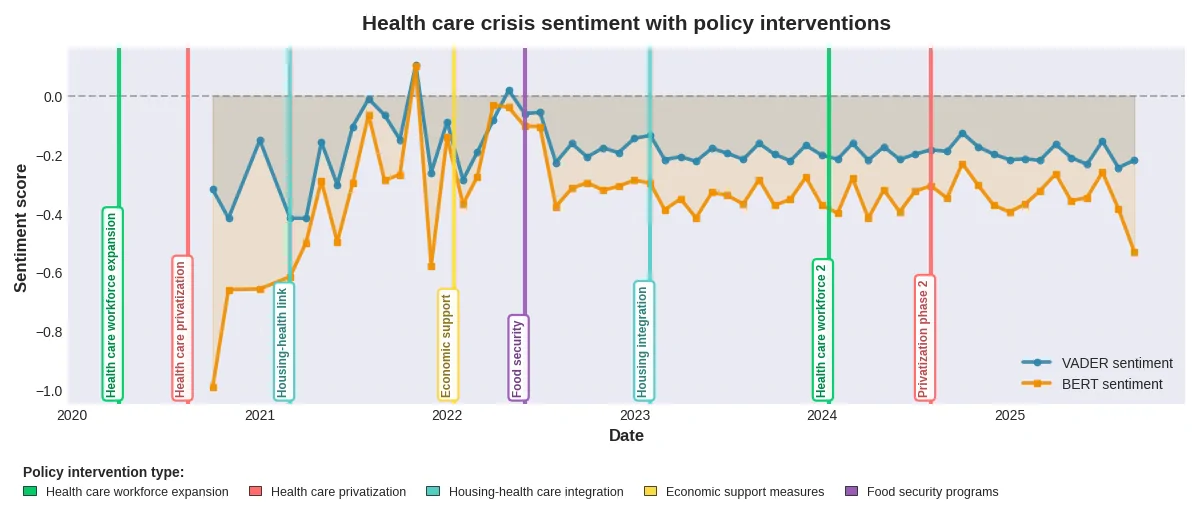
Time series data representing monthly BERT (orange) and VADER (blue) sentiment scores from October 2020 through September 2025 with an overlay of each of the 8 policy interventions assessed using a vertical color-coded line. The colors are used to designate the type of intervention. As can be observed by visual comparison of the 2 models in this figure, neither model demonstrated any significant shift resulting from any of the policy interventions. Given its sensitivity to contextual considerations, BERT was found to consistently produce lower (ie, more negative) values compared to VADER. Shaded areas extend from each series to zero. A legend that identifies each of the 5 types of policy intervention (health care workforce expansion, health care privatization, housing–health care integration, economic support measures, and food security programs) is provided at the bottom of the figure. BERT: bidirectional encoder representations from transformers; VADER: Valence Aware Dictionary and Sentiment Reasoner.

### Predictive Feature Analysis

The primary factor that predicted the expression of a negative sentiment about health care issues was the text length, that is, how much text there was in the news article (0.521). The second largest set of predictors were “calendar month” (0.244) and “year” (0.124). Based on topic indicators related to health care, the top indicators for new articles with high levels of information included “economic determinants of health” (0.038); “social inequality health effects” (0.022); “housing as health determinant” (0.019); “food insecurity health impact” (0.017); and “health care system crisis” (0.015). To test the sensitivity of our results, we also calculated SHAP values [24] for the same Random Forest Model; text length remained the dominant predictor under both methods. However, SHAP ranked the Economic Determinants of Health topic indicator above the calendar-based features (month and year), which is contrary to those found using Gini-based ranking. This difference is expected given the previously cited propensity of Gini Impurity to preferentially rank high cardinality continuous variables over less frequent or rare binary variables [25]. The full comparison is provided in Multimedia Appendix 2.

### Integrated Surveillance Dashboard

The results of all the analyses reported in the preceding Results subsections (Sentiment Surveillance through Predictive Feature Analysis) were synthesized into a prototype health care crisis monitoring dashboard (Figure 3). This dashboard demonstrates the operational capability of this study for performing near-real-time health system infoveillance. This prototype dashboard has 4 components.

At the top left is a donut chart illustrating the relative proportion of media content related to crisis themes in the last 3 months. This allows for a quick determination of what thematic area is currently the most prominent in the media environment.

At the top right is a multiseries line chart showing the time trend of temporal sentiments for each theme separately over the last 3 months. This will allow an analyst to determine if there are any themes that are diverging from the overall trend.

At the bottom left is a gauge showing the rolling mean sentiment score (–0.197, SD 0.410; n=142 headlines) of coverage of health care issues over the last 3 months. The gauge provides a single-number summary of the current state of health system infoveillance, as well as an immediate visual signal that the system has been negatively oriented over the past 3 months.

At the bottom right is a grouped bar chart showing topic volume by month over the past 6 months. This will enable an analyst to determine whether any themes are increasing their volumes faster than other themes in near real-time.

Together, these charts demonstrate how the proposed infoveillance can be turned into a decision-support tool for health policymakers. It does so by integrating all dimensions of analytical results—sentiment analysis, topic prevalence, and temporal dynamics into 1 display: infoveillance. This would enable policymakers to monitor ongoing developments without repeatedly conducting manual analysis. The current reading of –0.197 (SD 0.410) for sentiment, indicating that coverage of Canadian health care is well into negative territory, is consistent with findings presented in the Sentiment Surveillance subsection of the Results section.

## Discussion

### Principal Findings

The integration of the described multidisciplinary NLP pipeline, including techniques to perform sentiment analysis, topic modeling, causal network analysis, and forecasting, to monitor health system failures in multiple health themes was demonstrated in this study to be feasible.

A rolling sentiment indicator for the last 3 months; a trendline for each health theme; and topic volume indicators were displayed on the same dashboard for the purpose of providing a decision support system for stakeholders. Furthermore, the dashboard will continue to be dynamic as new headline data continues to automatically feed into it. As such, this study extends the infodemiology framework by Eysenbach [10] and the digital disease detection paradigm by Brownstein et al [13], originally developed for detecting outbreaks of infectious diseases, to detecting structural failures in health care systems. A polycrisis represents a great opportunity to test the proposed surveillance tool to identify cross-theme dynamics and to assess policy impacts across different health themes.

Additionally, the dual model sentiment approach achieved high levels of accuracy (accuracy=0.97), which are consistent with other research findings that indicate that short crisis-theme–specific texts can provide a relatively clear lexical valence for both rule-based and transformer models [26]. It is also possible that the high degree of agreement between the 2 models was influenced to some extent by label inheritance because the training data used for BERT were labeled using VADER-derived labels [27]. Therefore, future surveillance systems would benefit from using human-annotated datasets to address the circularity issue. When there are resource constraints, the complementary strengths of the VADER (computationally efficient) and BERT (contextually sensitive) models may be used together [11]. This circularity is an acknowledged weakness in pipelines using lexical methods to develop training labels for subsequent transformation of a model, so the good correspondence between VADER and BERT can reasonably be interpreted as demonstrating internal consistency rather than independently validating the accuracy of either model against true values [28,29].

### Empirical Evidence of Polycrisis Dynamics

To date, there have been no studies that examine polycrisis systems in Canada using the Granger causality technique within a broad statistical framework to understand how and why various crises become linked. The results from the Granger causality analysis provide the first empirical, quantifiable description of polycrisis topology for discussions related to health care in Canada’s public discourses. One major finding relates to the strength of the relationship between food insecurity health impact discourse influencing coverage of housing as a health determinant discourse (*P*_FDR_=.002). As such, an increase in Canadian news coverage of food insecurity generally leads to an increase in coverage of housing as a health determinant in the subsequent month. This causal connection directly aligns with the polycrisis mechanism of causal entanglement by Lawrence et al [3] and supports the Cascade Institute’s assertion that cascading from “upstream” to “downstream” represents the distinguishing feature of a polycrisis [5].

In addition to this relationship, there also exists a relationship between coverage of economic determinants of health and food insecurity health impact discourse (*F*_1,54_=7.601, *P*_FDR_=.024), which suggests that an upward cascade occurs in the media coverage: coverage of economic hardship occurs before and predicts coverage of food insecurity, which then causes an increase in coverage of housing and social inequality issues. This does not indicate a causal chain among the underlying social conditions themselves—instead, intuitively monitoring economic discourse could provide some degree of early warning regarding whether downstream food-insecurity, housing, and social-inequality discourse will occur.

Health care system crisis discourse constitutes a separate causal branch and independently Granger-causes coverage of both housing as a health determinant and social inequality as a health issue (Table 3). Thus, rather than occurring along a single linear chain, Canada’s health care polycrisis discourse appears to propagate via 2 independent, parallel upstream pathways.

While food insecurity health impact was not the largest singular theme (Table 1), it functioned as the central node of the lag-1 causal network (Table 3): food insecurity health impact received a Granger signal from economic determinants of health (*F*_1,54_=7.601) and, in turn, Granger-caused coverage of both housing as a health determinant and social inequality as a health issue—the 2 strongest directional connections noted in this study. This affirms Dietitians Canada’s perspective on food insecurity as a cross-cutting determinant of health [30]; additionally, it indicates that food insecurity acts as a discourse pathway through which economic hardship is transformed into coverage of housing and social-inequality health concerns.

Health care system crisis discourse occupied an upstream position in the lag-1 causal network: it independently Granger-caused coverage of housing and social inequality, and it did not receive any significant input signal from any other theme (Table 3). At longer lags (Figure 5), it received one significant input, from economic determinants of health at lag 6. Overall, collectively, these findings demonstrate an empirical implementation of polycrisis theory in relation to nation-states in a way that has not occurred previously either theoretically or worldwide [31].

### Policy Surveillance: Null Signal With Implications

The fact that we did not observe a statistically significant effect from any of our policy interventions on the media sentiment variable (combined with consistently low effect sizes for those that did exhibit some type of impact) indicates that these results could be valuable in terms of assessing the efficacy of health policies. In summary, while the absence of statistically significant effects indicates that none of our policy interventions produced a sufficient shift in the media’s prevailing negative discourse to produce observable changes in media surveillance signals, our results are consistent with evidence suggesting that during periods where there are sequential, multiple crises occurring at the same time, the cumulative nature of discrete, policy-based announcements continues to fail to alter the aggregate context of the discourse space [32]. Further, it is worth noting that the only policy initiative—health care privatization—that has the largest implementation level (0.8) exhibits a relatively short time frame (1 day) for retaining any type of influence. This short time frame may be attributed to ideologically polarizing announcements generating positive and negative coverage simultaneously, thereby cancelling each other out in total media sentiment. Additionally, the fact that text length and calendar year were stronger predictors of negative sentiment than any topic indicator suggests that the polycrisis itself represented the primary surveillance signal throughout the examined time frame and subject matter area. However, it should also be noted that text length is a stylistic or editorial aspect of headline writing and not a substantive measure of crisis severity. Therefore, the dominance of text length as a predictor of negative sentiment may reflect differences in headline lengths across outlets or over time instead of being an actual representation of a Surveillance signal.

### Future Directions

The focus of this study on news headlines only limits the analysis to the professional media layer of public discourse about Canada’s health care polycrisis and does not include the immediate, direct, and personal reactions of the public. Additional data layers through social media platforms such as Reddit or X could expand upon the methodology described in this study to survey public opinion directly during a comparable public health crisis [33]. Developing alternative methodologies to compare the use of the LDA topic model used throughout this study to other embedding-based models such as BERTopic and Top2Vec, which are capable of extracting local and coherent topics from short informal text, is another potential area of inquiry for subsequent studies.

Further possibilities exist for enhancing this analytical pipeline using large language models (LLMs). For example, a general-purpose LLM such as ChatGPT (OpenAI) may be trained to identify sentiment, determine themes, or provide summaries regarding the relevance of policy statements to specific stories with relatively minimal coding. A comparative study examining the feasibility of applying LLMs for identifying stance in public health discourse identified that all LLMs carry a significant amount of risk associated with misclassifying content relative to human-annotated expert standards. Only a few well-prompted models produced results comparable to those obtained via human annotation [34]. Therefore, if LLMs are used in place of the machine learning classifier used in the current study, they will most likely sacrifice precision and reproducibility (ie, prompt sensitivity, model version drift, and lack of a fixed audited lexicon similar to VADER’s or an audited accuracy similar to the BERT model’s) for increased ease-of-use and speed. These trade-offs should be evaluated empirically instead of being assumed. Since the topic and sentiment labels developed through LDA and VADER were used in the current study, substituting or supplementing them with an LLM-based classifier provides a clear direction for a future, focused-methodology-oriented study. We chose not to do so because it would alter the entire pipeline of analyses supporting the conclusions drawn from this study as opposed to improving upon it.

### Limitations

Headlines in news articles are an example of how a particular news organization can construct its interpretation of what is important or “newsworthy.” Therefore, while the data collected through our analysis may reflect a consensus of what was considered most newsworthy at a given time, it does not necessarily reflect directly stated opinions from members of the general population. Although we cannot reproduce all instances of news article collection (ie, we could not replicate every single news headline retrieved using the Google News API), the fact that we have access to archives of all previously retrieved headlines can allow us to analytically reproduce our results. We also recognize that by relying on keyword-based methods to determine which themes were represented in our dataset, we may have artificially increased the number of occurrences of certain categories of topics (eg, “health care system crisis”). As such, there may be some degree of bias introduced into our analyses due to overcounting of specific topic areas. BERT relies upon VADER-derived labels as part of its model development process. This reliance limits the extent to which independent models can be compared to one another. Given that we only analyzed 8 separate policy interventions in this study, there may be limitations associated with detecting even relatively small effects of these policies based solely on statistical analysis alone. Our corpus only includes English-language sources; therefore, if French-language coverage exists in Quebec or Acadia, it would not be included in our analysis. Future studies using this methodology could expand the types of languages represented in their corpora, extend the length of the policy window examined in each case study, and use human-annotated training labels instead of VADER-labeled training labels.

Although we were able to track Canada’s polycrisis discourse signal across 259 different news outlets, there is evidence that the discourse signal has been concentrated among a few large national news outlets. Specifically, the top 5 news outlets accounted for 26.8% (1809/6757) of all headlines, and the top 10 news outlets accounted for 37.8% (2554/6757) of all headlines. Additionally, the news outlet with the most contributions to the discourse signal was the CBC, which contributed to 13.3% (897/6757) of the total. Editorial positioning and regional focus of the larger news outlets may have had a greater influence on the discourse signal compared to a more even distribution of samples from smaller news outlets. However, we did not classify each news organization into categories based on political leanings, credibility as determined by third parties, or audience size. Descriptively, the news outlet pool included a variety of institutional positions, such as national broadcast networks (eg, CBC, CTV News, and Global News), financial-oriented publications (eg, Financial Post), policy institutes focused on markets (ie, Fraser Institute), and labor- or progressive-oriented organizations (eg, Canadian Union of Public Employees and Canadian Centre for Policy Alternatives). As such, we cannot exclude the possibility that varying amounts of reporting will occur from differing types of news outlets or orientations. Future studies should include classification and weighting of outlet-level characteristics.

Similarly, we did not compare the media-derived surveillance signal to an independent measure of system stress. A valid method of assessing whether the media-derived surveillance signal reflects true levels of system stress would be through comparison to real-time measures of health care usage metrics (such as Emergency Department Wait Times, Hospital Occupancy Rates, etc). Therefore, creating a link between the media-derived surveillance signal and an objective measure of system stress will be an important next step before using the model as an operational tool.

Lastly, because our media-derived surveillance signal represents only the frames chosen by media organizations, we capture what they choose to report about but do not capture what Canadians think about the polycrisis. Therefore, integrating this media-based signal with either publicly available social media or survey data representing Canadian public opinion would provide insight into how closely media framing aligns with the perceptions of Canadians regarding the polycrisis.

### Conclusions

This study introduces a multimethod NLP-based surveillance pipeline which represents the first computational characterization of Canada’s health care polycrisis as a causally entangled discourse system. Through the analysis of 6757 news headlines in conjunction with 5 interdependent health crisis themes, the pipeline found that negative sentiment has been consistently represented in media coverage throughout this study sample period. The study also demonstrated significant Granger-causality pathways among the health crisis themes consistent with polycrisis dynamics and indicated that none of the current policy interventions have produced a detectable footprint in the overall discourse signal.

These findings provide three principles to apply to health informatics in terms of designing surveillance systems for polycrisis: (1) health informatics pipelines designed to collect data from multiple health themes will be required to monitor cross-theme causal relationships missed by single-topic systems, (2) conducting Granger-causality tests among discourse themes will allow for identifying potential leading-indicators within each health theme to support development of early-warning alerting capabilities, and (3) assessing the impact of policies through health informatics surveillance systems should use FDR-corrected statistical inference and report effect sizes to preclude misinterpretation of policy effects as effective.

Although the 12-month forecasts are uncertain (all 95% CIs include zero) and show a rise only for health care system crisis; the cross-theme dynamics observed over 5 years of coverage indicate that health informatics infrastructure capable of analyzing polycrisis dynamics at a large scale is now a functional necessity rather than merely a methodological aspiration.

## Appendix

Multimedia Appendix 1 Policy interventions assessed for impact on media sentiment. All 8 policy interventions considered in this study, including the 3 that predated sufficient headline volume for statistical testing (marked with an asterisk) and were therefore excluded from the results reported in Table 4.

Multimedia Appendix 2 Random forest feature importance: Gini Impurity vs SHAP (Shapley additive explanations). Comparison of feature importance rankings for the random forest classifier (negative-sentiment prediction) using the default Gini impurity measure versus mean absolute SHAP values.

Multimedia Appendix 3 Raw dataset of news headlines. The complete dataset of 6757 news headlines analyzed in this study, with crisis type, headline text, publication date, and source outlet recorded for each headline.

Multimedia Appendix 4 Analysis code and reproducibility package. The complete Python analysis code (data collection, preprocessing, topic modelling, sentiment analysis, time-series forecasting, and Granger causality testing) used to produce every table and figure in this manuscript, together with the topic-label correction script applied during manuscript revision and the raw output files each stage produces.

## Abbreviations

ARIMA: autoregressive integrated moving average
BERT: Bidirectional Encoder Representations from Transformers
CBC: Canadian Broadcasting Corporation
ETS: error, trend, and seasonality
FDR: false discovery rate
LDA: latent Dirichlet allocation
LLM: large language model
NLP: natural language processing
NLTK: Natural Language Toolkit
SHAP: Shapley additive explanations
VADER: Valence Aware Dictionary and Sentiment Reasoner

## Data Availability

The raw data set containing the 6757 news headline titles analyzed within this study was obtained through the use of the GoogleNews Python library (v1.6.14), using the Google News API. We report in detail in the methods section all of our search terms, date ranges, and exclusion criteria, which can be used to replicate how we collected the dataset. A detailed description of all 12 of the search query terms we used to create the overall corpus of news headlines is available in the Methods (Data Collection) section, so that readers may independently reproduce our dataset. The same CSV file we have used to examine the data in this study, which includes the crisis type, headline text, publication date, and source outlet information for all 6757 headlines, has been made available as Multimedia Appendix 3 to accompany this submission. The Python analysis scripts we used to analyze the data (VADER [Valence Aware Dictionary and Sentiment Reasoner], BERT [Bidirectional Encoder Representations from Transformers] fine-tuning, latent Dirichlet allocation, ARIMA [autoregressive integrated moving average], ETS [error, trend, and seasonality], and Granger Causality), along with the table outputs produced by each script, have been included in Multimedia Appendix 4 accompanying this submission. Therefore, all numbers reported in this paper can be recalculated directly.
