# Supplementary material for: Monitoring Canada’s Health Care Polycrisis Through News Headline Analysis: Infodemiology Study
Source: JMIR Infodemiology. 2026 Oct 29;6:e99655. doi: 10.2196/99655 (PMC13760331; doi:10.2196/99655)
Supplement: Supplementary file 1 [file infodemiology_v6i1e99655_app1.docx]

| **Intervention** | **Date** | **Type** | **Implementation Score** | **Description** | **Statistical Testing Status** |
| --- | --- | --- | --- | --- | --- |
| Healthcare Workforce Expansion (1)* | 2020-04-01 | Healthcare Workforce Expansion | 0.75 | Emergency hiring of healthcare workers | Excluded: date precedes the corpus start date (2020-10-17). |
| Healthcare Privatization (1)* | 2020-08-15 | Healthcare Privatization | 0.30 | Private healthcare expansion initiatives | Excluded: date precedes the corpus start date (2020-10-17). |
| Housing-Health Link* | 2021-03-01 | Housing-Healthcare Integration | 0.45 | Housing as health determinant programs | Excluded: fewer than 10 headlines in the pre-intervention window (3 pre / 13 post). |
| Economic Support | 2022-01-15 | Economic Support Measures | 0.20 | Economic support for vulnerable populations | Included in Table 4. |
| Food Security | 2022-06-01 | Food Security Programs | 0.40 | Food security and nutrition programs | Included in Table 4. |
| Housing Integration | 2023-02-01 | Housing-Healthcare Integration | 0.55 | Integrated housing-healthcare services | Included in Table 4. |
| Healthcare Workforce Expansion (2) | 2024-01-15 | Healthcare Workforce Expansion | 0.60 | Healthcare workforce retention programs | Included in Table 4. |
| Healthcare Privatization (2) | 2024-08-01 | Healthcare Privatization | 0.80 | Expanded private healthcare services | Included in Table 4. |
