# Supplementary material for: Monitoring Canada’s Health Care Polycrisis Through News Headline Analysis: Infodemiology Study
Source: JMIR Infodemiology. 2026 Oct 29;6:e99655. doi: 10.2196/99655 (PMC13760331; doi:10.2196/99655)
Supplement: Supplementary file 2 [file infodemiology_v6i1e99655_app2.docx]

| **Feature** | **Gini Importance** | **Mean \|SHAP\|** | **Gini Rank** | **SHAP Rank** |
| --- | --- | --- | --- | --- |
| text_length | 0.521 | 0.042 | 1 | 1 |
| topic_Economic Determinants of Health | 0.038 | 0.022 | 4 | 2 |
| month | 0.244 | 0.016 | 2 | 3 |
| year | 0.124 | 0.016 | 3 | 4 |
| topic_Housing as Health Determinant | 0.019 | 0.011 | 6 | 5 |
| topic_Social Inequality Health Effects | 0.022 | 0.009 | 5 | 6 |
| topic_Food Insecurity Health Impact | 0.017 | 0.007 | 7 | 7 |
| topic_Healthcare System Crisis | 0.015 | 0.006 | 8 | 8 |
